# Patients’ Concerns About Receiving Preemptive Pharmacogenomic Testing: Results from a Large, Longitudinal Survey of RIGHT Study Participants

**DOI:** 10.3390/jpm15060258

**Published:** 2025-06-17

**Authors:** Joel E. Pacyna, Suzette J. Bielinski, Janet E. Olson, Richard R. Sharp

**Affiliations:** 1 Biomedical Ethics Research Program, Mayo Clinic, Rochester, MN 55905, USA; pacyna.joel@mayo.edu; 2 Department of Health Sciences Research, Mayo Clinic, Rochester, MN 55905, USA; 3 Center for Individualized Medicine, Mayo Clinic, Rochester, MN 55905, USA

**Keywords:** pharmacogenomics, patient concerns, return of results, drug selection, drug dosing

## Abstract

**Background:** As more healthcare institutions consider providing preemptive pharmacogenomic (PGx) testing to greater numbers of their patients, it will be important to consider the potential concerns patients may have about the generation of preemptive PGx information. To date, few studies have examined the nature and incidence of patient concerns about preemptive PGx testing. **Methods:** We conducted a longitudinal survey study of 5000 patients receiving preemptive PGx testing in the Mayo Clinic RIGHT study. We assessed patient concerns regarding issues of data confidentiality, cost implications, comprehension of results, and potential disruption of pre-existing medication regimens. Participants were surveyed before and after they received PGx results from the RIGHT study. **Results:** We achieved 92.8% and 74.4% response rates on the pre- and post-results surveys, respectively. Participants had low levels of concern about PGx testing overall. However, 25.5% of participants were “quite/extremely concerned” about insurance implications, and 30.1% were “quite/extremely” concerned about increased out-of-pocket costs for prescription medications that might result from PGx testing. These same concerns were significantly reduced on the post-results survey. Patients who initially expressed concerns regarding their ability to understand PGx results were more likely to report having difficulty understanding results on the post-results survey. **Conclusions:** Our findings suggest that as healthcare institutions look to increase preemptive PGx screening, attention should be given to potential concerns patients may have around such testing. Educational interventions aimed at supporting patient understanding of PGx results and addressing potential concerns will be important elements of a successful PGx program.

## 1. Introduction

Pharmacogenomic (PGx) testing is a genetic test that evaluates specific genes associated with how the body responds to prescription drugs. Physicians may use information generated by PGx testing as they consider which prescription drugs and drug doses are best suited to their patients. Several healthcare institutions have begun to focus on pharmacogenomics (PGx) as a potential candidate for broad implementation [1,2,3]. This is not surprising, as PGx testing has the potential to address some of the perennial challenges facing healthcare institutions with respect to prescription medications, including the challenge of reducing adverse drug reactions and achieving optimal drug selection and dosing more quickly [4]. However, despite the appeal of broadly implementing PGx screening, its success will require the navigation of implementation challenges that come with scale, not the least of which is addressing potential concerns patients may have about the generation of PGx information. For example, although clinicians routinely counsel patients about drug selection and dosing, broad implementation of preemptive PGx must not over-complicate clinician–patient conversations about prescription medications and must not generate a substantial number of new patient concerns. In another example, although institutions may see preemptive PGx information as valuable for creating individualized strategies for the management of disease, broad implementation should not proceed on the assumption that patients will be unconcerned about the impact that PGx information might have on future opportunities for medical intervention or insurability.

While some empirical data suggest that patients find PGx information to be acceptable and important [5,6,7,8,9,10], few studies have examined potential patient concerns [11]. Although there is a general acknowledgment that support structures for genomic screening programs must adapt to accommodate the large volumes of patients they will serve [12,13,14], there is a critical need to characterize the nature and prevalence of patients’ concerns about preemptive PGx testing. Failure to do so will result in lost opportunities to design targeted interventions that address these concerns and support needs. Worse still, failure to address patient concerns in large implementation projects could compromise patients’ trust in healthcare institutions and could lead to skepticism about the role and value of genetics in healthcare.

To begin to address the need for data on the nature and incidence of patient concerns in the context of a preemptive PGx implementation project, we conducted a longitudinal survey study of 5000 patients who volunteered to receive a large, preemptive PGx panel in the context of a translational PGx research study within Mayo Clinic’s primary care practice. One of our aims was to assess patients’ concerns before and after receiving PGx results from the study. Our findings illuminate patient concerns and point to potential support needs that may arise as institutions seek to expand the use of preemptive PGx testing.

## 2. Methods

### 2.1. Setting and Participants

The RIGHT study provided a pharmacogenomic panel to 10,085 individuals [15,16]. Individuals invited to the RIGHT study were participants in the Mayo Clinic Biobank, and most received care in one of Mayo Clinic’s local primary care practices. Inclusion criteria for the RIGHT study required participants to be 18 years of age or older and able to provide written informed consent. Because inclusion criteria did not require clinical indication for the testing, the pharmacogenomic panel was considered “preemptive”. Participants were invited by mailed letter along with a study brochure and consent form and were instructed to return their signed written consent in a self-addressed, postage-paid envelope to enroll in the study. Participation involved consenting to receive a preemptive PGx panel from a CLIA-certified laboratory, with PGx results documented in participants’ electronic health records and made available to their primary healthcare providers in the Mayo Clinic health system. Recruitment to the parent study occurred from May 2016 to August 2017. From participants who consented to participate in the RIGHT study, we randomly selected 5000 individuals (roughly half the RIGHT study sample) to receive the survey before they received their results. RIGHT study participants who were known to be enrolled in another large genomic implementation study being conducted at Mayo Clinic were excluded from our survey sampling frame in order to avoid the possibility of participants conflating the two study experiences as they responded to our survey questions.

All participants received PGx results in a mailed packet. This mailing contained a cover letter and a report generated by OneOme (Minneapolis, MN, USA) [17]. The OneOme report, which was designed to be a provider-facing report, supplied individualized information about patients’ PGx results, including an extensive list of common medications categorized into bins of potential risk for drug–gene interactions. Therapeutic recommendations were available for over 30 of these medications at the time that PGx results were returned [15]. The OneOme report also contained detailed information about patients’ drug metabolism profiles and a bibliography of current literature supporting the conclusions in the report. The OneOme report was also included in participants’ electronic health records.

### 2.2. Surveys

We designed two surveys, the first of which was administered before participants received their PGx test results; the second survey was administered after participants received results. The first survey (from now on referred to as the “pre-results survey”) was a 64-item survey that included items from validated measures from the behavioral health literature along with standard demographic questions. The second survey was administered after participants received their PGx results in the mail. This second survey (from now on referred to as the “post-results survey”) was a 50-item survey that repeated several measures from the pre-results survey and included additional items focused on participant experiences receiving PGx results.

### 2.3. Repeated Measures

Participant concerns assessed before and after receiving PGx results were assessed using two sets of questions, each containing three items. The first set of questions was presented to all respondents and included the following items: “How concerned are you that your test results may not stay confidential?”, “How concerned are you that your test results may be difficult for your doctor to understand?”, and “How concerned are you that your test results may make you ineligible for insurance coverage for certain prescription drugs?” Respondents rated their concern for each item on a four-point scale (“Extremely concerned,” “Quite concerned,” “Slightly concerned,” and “Not at all concerned”). Using skip logic, we presented the second set of three questions only to respondents who had indicated previously that they were taking one or more prescription medications. This second set of questions used the same four-point scale as the first set to rate respondents’ level of concern for each item: “How concerned are you that your test results might show that you need to switch to a different prescription drug?”, “How concerned are you that your test results might show that a prescription drug with a higher out-of-pocket cost would work better for you?”, “How concerned are you that your test results might show that you need a higher dose of a prescription drug you are currently taking?”, and “How concerned are you that your test results might show that you need a lower dose of a prescription drug you are currently taking?”

### 2.4. Additional Measures

Other variables collected in the pre-results survey included respondents’ self-reported number of active prescriptions, self-reported health [18], self-reported health literacy [19], and previous experience with genetic testing and PGx testing. In the pre-results survey, we also asked participants how concerned they were about being able to understand their results (“Extremely concerned,” “Quite concerned,” “Slightly concerned,” and “Not at all concerned”). Variables collected in the post-results survey included items querying respondents about whether they had questions about their results and whether they understood them.

### 2.5. Data Collection

We began fielding the pre-results survey in March 2017. Post-results surveys were distributed beginning in November 2018, following a mailed return of participants’ PGx results. Surveys were mailed in a printed, full-color booklet along with a cover letter and postage-paid return envelope. The Mayo Clinic Survey Research Center processed incoming responses and tracked participation. Non-responders to the first mailings of each survey were mailed a reminder survey. Completed survey responses were double-entered by trained data-entry staff. Ambiguities in the survey responses were flagged, and a study team member (JP) reviewed each flag as part of the data cleaning process. When the intent of the respondent could be discerned, the data set was updated; inscrutable responses were marked as missing data.

We supplemented the self-reported demographic and psychosocial data collected in the survey with information abstracted from respondents’ electronic health records and from documented responses to an intake questionnaire that respondents completed at the time they enrolled in the Mayo Clinic biobank. These data included participant age, sex, and education level.

### 2.6. Data Analysis

Data were analyzed using R version 4.0.4 (R Foundation for Statistical Computing, Vienna, Austria). Frequencies and percentages were calculated for categorical variables, and means and standard deviations were calculated for continuous variables. Responses to the concern questions were compared and ranked by prevalence in the sample. Chi square tests and two-sample *t* tests were used, as appropriate, to compare participants’ responses before and after receiving their PGx results. *p*-values of 0.05 or lower were considered statistically significant.

## 3. Results

Of the 5000 individuals who were randomly sampled from participants in the RIGHT study, 17 were not included due to death, participation preferences, or study eligibility issues identified after the initial sampling was created. We mailed pre-results surveys to the remaining 4983 individuals and received 4624 completed surveys back from study participants (92.8% survey completion rate). Post-results surveys were sent to participants who completed the pre-results survey and had subsequently received their PGx results in the mail. Of the 4624 pre-results survey respondents, 45 were deceased before the post-survey mailing, and 1 was deemed ineligible, resulting in 4578 individuals available to receive the post-results survey. Of this group, 3408 returned completed surveys (74.4% survey completion rate).

Table 1 provides demographic information and a comparison of responders and non-responders for both surveys. For the pre-results survey, respondents were older (63.7 years vs. 57.0 years, *p* < 0.001) and more educated (*p* = 0.021). More responders were white (97.5% vs. 92.2%, *p* < 0.001), non-Hispanic (97.6% vs. 95.0%, *p* = 0.010), and married (76.3% vs. 70.2%, *p* = 0.031) compared to non-responders. For the post-results survey, respondents were younger (63.1 years vs. 64.9 years, *p* < 0.001) and more educated (*p* < 0.001), and more were female (66.7% vs. 62.1%, *p* = 0.004), white (97.8% vs. 96.7%, *p* = 0.038), non-Hispanic (97.9% vs. 96.8%, *p* = 0.046), and married (77.8% vs. 71.8%, *p* < 0.001).

Table 2 summarizes participants’ responses—before and after receiving PGx results—to survey items about potential concerns about the impact of PGx results. While participants expressed low levels of concern about the generation of PGx information, participants expressed the greatest amount of concern regarding future increases in out-of-pocket costs for medications (30.1% were “quite concerned” or “extremely concerned”) and insurance eligibility (26.5% were “quite concerned” or “extremely concerned”). Participants also expressed concerns about the potential need to switch medications or change doses of existing medications (for both items, 17.2% were “quite concerned” or “extremely concerned”). Less common were concerns about the confidentiality of PGx results (10.0% were “quite concerned” or “extremely concerned”) and concerns about their clinician’s ability to understand PGx results (6.3% were “quite concerned” or “extremely concerned”).

On the post-results survey, there was a statistically significant reduction in concern for all the survey items pertaining to the potential impact of PGx results, with the exception of concerns about the ability of physicians to understand the results. Most notably, participants’ concerns about future medication costs were much lower (8.8% were “quite concerned” or “extremely concerned”, down from 30.1% of respondents in the pre-results survey), as were concerns about insurance eligibility (12.2% were “quite concerned” or “extremely concerned”, down from 26.5%). Of the concerns measured before and after participants received their PGx results, concern about future insurance eligibility remained the greatest concern after receipt of results, with 12.2% of participants indicating that they were “quite concerned” or “extremely concerned” about the potential impact their PGx results might have on future health insurance coverage.

Table 3 compares a subgroup of respondents who reported being “quite concerned” or “extremely concerned” about insurability after receiving results with the remainder of respondents only “slightly concerned” or “not at all” concerned about insurability after receipt of results. Those who were “quite concerned” or “extremely concerned” were older (66.3 years vs. 62.4 years, *p* < 0.001), less educated (*p* < 0.001), reported poorer health (*p* < 0.001), and were more likely to be unsure about whether they had previously received PGx testing (*p* = 0.001). Males (*p* < 0.001), non-white participants (*p* < 0.001), and participants reporting inadequate health literacy (*p* = 0.024) were more likely to report being “quite concerned” or “extremely concerned” about insurance eligibility. Participants who reported having employer-provided health insurance (*p* < 0.001) were less likely to be “quite concerned” or “extremely concerned”, while participants reporting having privately purchased health insurance (*p* = 0.002) or government-sponsored insurance (*p* < 0.001) were more likely to report being “quite concerned” or “extremely concerned” about insurance eligibility.

Although participants expressed low levels of initial concern (before receiving PGx results) about being able to understand their PGx results, 43.3% of respondents to the post-results survey reported not understanding their results, and 45.1% reported having questions about their results. Table 4 examines the relationship between the pre-results concerns participants had about being able to understand their PGx results and their reported ability to understand their PGx results after they had received them. Participants who reported having questions about their results and participants who did not report understanding their results were more likely to have anticipated these concerns at the time they completed the pre-results survey (*p* < 0.001).

## 4. Discussion

Our results suggest that patients have concerns about the generation of PGx information. Much of the literature on PGx implementation has focused, to date, on potential system-level barriers to implementation, including how PGx information will be stored and utilized in the context of an electronic health record or how clinical decision support tools might be designed to support prescribing practices. Prior studies have assessed patient knowledge in large implementation projects [1,20,21,22,23]. Other studies have examined provider receptivity to using PGx testing and the self-reported preparedness of pharmacists and prescribers. While these prior studies consider critical aspects of institutional preparedness for PGx testing, our data suggest that in addition to these considerations, successfully implementing PGx testing will require sensitivity to the potential concerns patients may have about the generation of PGx information. Our results suggest that several considerations are of considerable importance.

First, we observed elevated concerns regarding potential cost and insurance implications for prescription medications. These findings are consistent with findings from other studies. For example, Lachance and colleagues queried 450 patients (175 of them “healthy”, the remainder of them receiving care for heart failure or organ transplant) about the acceptability of PGx testing and found that many participants were concerned about future insurance issues (>60%) or workplace discrimination (>23%) [24]. Similarly, Lee and colleagues surveyed 120 individuals in an inner-city context and found that some had concerns about insurance coverage for the test (4.3%) [22]. In a qualitative study conducted in the UK, some general practice physicians aired concerns about potential “insurance loading”, in which PGx information available in the medical record could lead to higher out-of-pocket costs to patients [23]. Gawronski and colleagues found that in a low-income population, the potential for the testing to cost them money was the greatest concern [25]. 

In our study, over 30% of participants currently taking prescription medications indicated they were “Extremely concerned” or “Quite concerned” about potential out-of-pocket costs resulting from the generation of PGx information. This finding suggests that some individuals may be attuned to some of the economic uncertainties that surround a large, system-wide implementation of preemptive PGx testing and may worry that the optimization of drug prescribing may result in higher out-of-pocket costs.

Second, our data suggests that many of our participants’ concerns, including concerns about potential cost and insurability implications, decreased considerably after participants reviewed their PGx results. This finding aligns with a recent study of primary care patients who received genomic screening, where various concerns expressed by patients shortly after they received results were decreased when measured again 6 months later [26]. Participants’ concerns about PGx testing expressed at baseline may be largely due to a lack of personal familiarity with PGx testing.

Third, our findings suggest that, while many patients do not have concerns about their ability to understand PGx results, those who do have concerns before receiving their results are more likely to have questions about their results and report an inability to understand their results after they have received them. Patients’ inability to understand their PGx results may limit their ability to identify whether their PGx information is being adequately incorporated into their care and could lead to uncertainty about whether PGx information will result in safer, more individualized prescribing. Furthermore, patients’ perceptions about the complexity of their PGx results may lead to uncertainty about their physicians’ ability to understand PGx results. While patients’ concerns about confidentiality, cost, and disruption all decreased after PGx results were returned, their concerns regarding their physicians’ ability to understand PGx results increased slightly.

Lastly, while our findings highlight the absence of wide-ranging concerns about PGx testing in a setting where PGx testing has been implemented broadly, they also point to pockets of concern that may be amenable to educational interventions. To date, there have been a few studies examining the impact of patient education materials or interventions, though none has focused on informing patients about the potential downstream impact of PGx information on healthcare costs or insurability [27,28,29,30]. As patients become increasingly aware of the cost implications of advanced medical technologies (including forms of genomic screening), educational interventions such as brochures and web content could speak directly to costs associated with PGx testing—not only the costs of the tests, but also the likelihood that PGx testing will result in more complicated reimbursement of healthcare expenditures and greater patient responsibility for healthcare costs.

### Limitations and Future Research Opportunities

Our findings should be interpreted in light of several important limitations. First, participants in the RIGHT study were recruited from a large academic medical center and may not be typical of patients who receive care in other healthcare settings. Additionally, participants were recruited from a biobank and had previously demonstrated a favorable disposition toward biomedical research and, correspondingly, may have less concerns about biomedical research (including genetic research) than individuals without experience as biobank contributors. Second, our participants reflected the demographic composition of southeast Minnesota—predominately white, non-Hispanic, and more educated than the general US population. Our findings are therefore not representative of more diverse communities in the US. Third, participants’ expressions of concern were limited to the questions presented in the surveys; it is possible that other prominent patient concerns were present among our participants but were not captured in the survey questions. Fifth, our survey was conducted several years ago, and it is possible that patient concerns about potential implications of preemptive PGx testing evaluated several years ago do not reflect the concerns patients may have today. This is especially true given the challenges experienced by patients and healthcare systems during the COVID-19 pandemic. It is also possible that patient concerns about the potential cost and insurance implications of preemptive PGx testing are greater today than they were when the survey study was conducted. Finally, we observed a significant drop-off in survey completion between the pre-results survey and the post-results survey. While survey completion rates are examined in Table 1 and shown to be associated with a number of demographic characteristics, it is also possible that the drop-off in survey completion between time points may in some way be related to the outcomes we have examined. The data we collected did not allow us to explore this possibility, and methods for controlling for this potential bias (for example, by conducting a paired comparison of pre/post survey responses using only participants who completed both survey instruments) would have required discarding a significant amount of data. Even if a bias exists, however, our conclusions regarding the potential benefit of additional support mechanisms for recipients of preemptive PGx testing would still seem to be supported by our data.

Future research should explore the feasibility and effectiveness of interventions designed to directly address potential patient concerns about the generation of PGx information. This should include the evaluation of actual out-of-pocket cost implications for patients and the design of educational content intended to allay unfounded concerns patients may hold about the cost ramifications of generating PGx information. Future research should also seek, where possible, to develop and test interventions in contexts where PGx testing is delivered to large cohorts of individuals. For interventions to be feasible, they must be deliverable in contexts where preemptive PGx testing is scaled up to include significant numbers of individuals in a comprehensive healthcare system. Future research should also explore potential concerns about PGx information in contexts that serve more diverse patient populations and in diverse healthcare settings (such as Federally Qualified Health Centers) that lack the resources of academic medical centers and university hospitals [31].

## 5. Conclusions

This study is one of the first to examine potential concerns that patients may have about PGx testing in the context of a large PGx implementation project. Our results suggest that healthcare institutions planning to implement preemptive PGx testing for larger numbers of patients should consider the potential concerns patients may have about the impact of generating and returning PGx information. Institutions should also consider developing educational interventions aimed at proactively addressing common patient questions, including questions related to downstream medication costs, that may result from PGx testing. If PGx testing is to be successful, it must not only inform prescribing practices but also give patients confidence that they are in fact receiving the right drug at the right dose.

## Figures and Tables

**Table 1:** Demographics and survey non-response analysis for pre- and post-return of preemptive pharmacogenomic test results.

		Pre-Results Survey			Post-Results Survey		
	Original Sample Frame (N = 4983)	Responded (N = 4624)	Did Not Respond (N = 359)	*p*-Value	Responded (N = 3408)	Did Not Respond (N = 1170)	*p*-Value
Age in years—Mean (SD)	63.2 (15.5)	63.7 (15.2)	57.0 (18.0)	<0.001 ^a^	63.1 (14.9)	64.9 (16.0)	<0.001 ^a^
	N (%)	N (%)	N (%)		N (%)	N (%)	
Gender				0.185 ^b^			0.004 ^b^
Female	3269 (66)	3022 (65)	247 (69)		2274 (67)	727 (62)	
Male	1714 (34)	1602 (35)	112 (31)		1134 (33)	443 (38)	
Race				<0.001 ^b^			0.038 ^b^
White	4839 (97)	4508 (97)	331 (92)		3331 (98)	1131 (97)	
Other	143 (3)	115 (3)	28 (8)		76 (2)	39 (3)	
Ethnicity				0.010 ^b^			0.046 ^b^
Not Hispanic/Latino	4854 (97)	4513 (98)	341 (95)		3335 (98)	1132 (97)	
Hispanic/Latino	54 (1)	47 (1)	7 (2)		28 (1)	19 (2)	
Other/Unknown	75 (2)	64 (1)	11 (3)		45 (1)	19 (2)	
Marital Status				0.031 ^b^			<0.001 ^b^
Married/Partnered	3781 (76)	3529 (76)	252 (70)		2652 (78)	840 (72)	
Not Married/Partnered	1201 (24)	1094 (24)	107 (30)		755 (22)	330 (28)	
Education				0.021 ^b^			<0.001 ^b^
8th grade or less	7 (0)	7 (0)	0 (0)		4 (0)	3 (0)	
Some high school	33 (1)	29 (1)	4 (1)		9 (0)	19 (2)	
High school graduate or GED	692 (14)	640 (14)	52 (15)		403 (12)	225 (20)	
Vocational/technical school	452 (9)	418 (9)	34 (10)		301 (9)	115 (10)	
Some college or Assoc. degree	1086 (22)	987 (22)	99 (28)		710 (21)	266 (23)	
Four-year college graduate	1319 (27)	1224 (27)	95 (27)		943 (28)	274 (24)	
Graduate/professional school	1302 (27)	1233 (27)	69 (20)		986 (29)	237 (21)	

a. Two-sample *t* test. b. Pearson’s chi-squared test.

**Table 2:** Participant concerns about preemptive pharmacogenomic testing before and after receipt of test results.

	Extremely Concerned N (Row %)	Quite Concerned N (Row %)	Slightly Concerned N (Row %)	Not at all Concerned N (Row %)	*p*-Value
Asked of all survey respondents					
Concerns about insurance eligibility					<0.001
Pre-ROR ^a^ survey	467 (10)	600 (13)	1630 (36)	1849 (41)	
Post-ROR ^b^ survey	147 (4)	260 (8)	955 (29)	1977 (59)	
Concerns about confidentiality					<0.001
Pre-ROR survey	156 (3)	301 (7)	1197 (26)	2902 (64)	
Post-ROR survey	42 (1)	120 (4)	659 (20)	2519 (75)	
Concerns about physician understanding					<0.001
Pre-ROR survey	84 (2)	199 (4)	778 (17)	3478 (77)	
Post-ROR survey	38 (1)	166 (5)	848 (25)	2284 (69)	
Asked only of survey respondents currently taking prescription medications					
Concerns about out-of-pocket costs					<0.001
Pre-ROR survey	415 (11)	693 (19)	1353 (37)	1219 (33)	
Post-ROR survey	66 (3)	126 (6)	374 (17)	1608 (74)	
Concerns about needing to switch medications					<0.001
Pre-ROR survey	177 (5)	455 (12)	1189 (32)	1859 (51)	
Post-ROR survey	27 (1)	107 (5)	534 (25)	1509 (69)	
Concerns about needing to change doses					<0.001
Pre-ROR survey	152 (4)	483 (13)	1274 (35)	1777 (48)	
Post-ROR survey	27 (1)	115 (5)	589 (27)	1448 (67)	

a. Pre-ROR = “Before return of PGx results”. b. Post-ROR = “After return of PGx results”.

**Table 3:** Characteristics of individuals who remain quite concerned or extremely concerned about insurance eligibility after receiving PGx results from the RIGHT study.

	Quite or Extremely Concerned About Insurance Eligibility			
	No (N = 2932)	Yes (N = 407)	Total (N = 3339)	*p*-Value
Age in years				<0.001 ^a^
Mean (SD)	62.4 (15.1)	66.3 (13.1)	62.9 (14.9)	
Range	22.1–83.0	27.0–82.8	22.1–83.0	
	N (%)	N (%)	N (%)	
Gender				<0.001 ^b^
Female	1991 (8)	239 (59)	2230 (67)	
Male	941 (32)	168 (41)	1109 (33)	
Marital status				0.340 ^b^
Divorced/Widowed/Separated/Single	636 (22)	101 (25)	737 (22)	
Married/Partnered	2295 (78)	306 (75)	2601 (78)	
Race				<0.001 ^b^
Other	56 (2)	19 (5)	75 (2)	
White	2875 (98)	388 (95)	3263 (98)	
Ethnicity				0.085 ^b^
Not Hispanic/Latino	2874 (98)	393 (97)	3267 (98)	
Other/Unknown	37 (1)	7 (2)	44 (1)	
Hispanic/Latino	21 (1)	7 (2)	28 (1)	
Self-reported health literacy ^c^				0.024 ^b^
Adequate health literacy	2696 (94)	359 (91)	3055 (94)	
Inadequate health literacy	170 (6)	35 (9)	205 (6)	
Education				<0.001 ^b^
High school graduate or less	320 (11)	77 (19)	397 (12)	
Vocational, technical, or business school	237 (8)	52 (13)	289 (9)	
Some college or Associates degree	618 (21)	78 (20)	696 (21)	
Four-year college graduate	839 (29)	93 (24)	932 (28)	
Graduate or professional school	879 (30)	96 (24)	975 (30)	
Self-reported health status				<0.001 ^b^
Excellent	547 (19)	43 (11)	590 (18)	
Very good	1405 (48)	179 (44)	1584 (48)	
Good	801 (27)	141 (35)	942 (28)	
Fair	155 (5)	34 (8)	189 (6)	
Poor	16 (1)	6 (2)	22 (1)	
Previous experience with Gx testing				0.078 ^b^
Yes	230 (8)	33 (8)	263 (8)	
No	2552 (87)	342 (84)	2894 (87)	
Not sure	146 (5)	31 (8)	177 (5)	
Previous experience with PGx testing				0.047 ^b^
Yes	41 (1)	5 (1)	46 (1)	
No	2745 (94)	368 (91)	3113 (94)	
Not sure	140 (5)	31 (8)	171 (5)	
Health insurance coverage				
No insurance	20 (1)	5 (1)	25 (1)	0.231 ^b^
Employer provided insurance	1654 (56)	175 (43)	1829 (55)	<0.001 ^b^
Privately purchased insurance	391 (13)	78 (19)	469 (14)	0.002 ^b^
Government program (e.g., Medicare)	1331 (45)	227 (56)	1558 (47)	<0.001 ^b^

a. Two-sample *t* test. b. Pearson’s Chi-squared test. c. Participants indicating they were “Extremely confident” or “Quite a bit confident” with filling out medical forms on their own were coded as having “Adequate” health literacy. Those who indicated they were “Somewhat confident”, “A little bit confident”, or “Not at all confident” with medical forms were coded as having “Inadequate” health literacy [19].

**Table 4:** Comparison of participants’ concerns about understanding their PGx results (before receiving them) with their reflections on their ability to comprehend them (after receiving them).

		I Had a Lot of Questions About My PGx Results ^b^			I Understood My PGx Results ^b^		
	Total (N = 3291)	Agree ^c^ (N = 1477)	Not Agree ^d^ (N = 1799)	*p* Value	Agree ^c^ (N = 1865)	Not Agree ^d^ (N = 1426)	*p* Value
How concerned are you that your test results may be difficult for you to understand? ^a^	N (%)	N (%)	N (%)	< 0.001 ^e^	N (%)	N (%)	<0.001 ^e^
Extremely concerned	51 (2)	35 (2)	15 (1)		15 (1)	36 (3)	
Quite concerned	266 (8)	168 (12)	96 (5)		87 (5)	179 (13)	
Slightly concerned	1485 (46)	716 (49)	771 (43)		756 (41)	729 (52)	
Not at all concerned	1447 (45)	540 (37)	893 (50)		988 (54)	459 (33)	

a. Asked of participants on the pre-results survey. b. Asked of participants on the post-results survey. c. Represents “Strongly agree” and “Agree” responses. d. Represents “Neither agree nor disagree,” “Disagree,” and “Strongly disagree” responses. e. Chi-square.

## Data Availability

The datasets presented in this article are not readily available. Requests to access the datasets should be directed to the corresponding author, Richard R. Sharp.

## References

[1] Dunnenberger H.M., Crews K.R., Hoffman J.M., Caudle K.E., Broeckel U., Howard S.C., Hunkler R.J., Klein T.E., Evans W.E., Relling M.V. (2015). Preemptive clinical pharmacogenetics implementation: Current programs in five US medical centers. Annu. Rev. Pharmacol. Toxicol..

[2] Wick J.A., Schmidlen T., Grande K., Moretz C., Ashcraft K., Green J., Moyer N., Blaxall B.C. (2023). Implementing comprehensive pharmacogenomics in a community hospital–associated primary care setting. J. Am. Pharm. Assoc..

[3] Wu A., Raack E.J., Ross C.J.D., Carleton B.C. (2024). Implementation and Evaluation Strategies for Pharmacogenetic Testing in Hospital Settings: A Scoping Review. Ther. Drug Monit..

[4] Weinshilboum R.M., Wang L. (2017). Pharmacogenomics: Precision Medicine and Drug Response. Mayo Clin. Proc..

[5] Lemke A.A., Hulick P.J., Wake D.T., Wang C., Sereika A.W., Yu K.D., Glaser N.S., Dunnenberger H.M. (2018). Patient perspectives following pharmacogenomics results disclosure in an integrated health system. Pharmacogenomics.

[6] Haga S.B., Mills R., Moaddeb J., Liu Y., Voora D. (2021). Delivery of pharmacogenetic testing with or without medication therapy management in a community pharmacy setting. Pharmacogenomics Pers. Med..

[7] Kaur G., Nwabufo C.K. (2024). Healthcare provider and patient perspectives on the implementation of pharmacogenetic-guided treatment in routine clinical practice. Pharmacogenetics Genom..

[8] Kusic D., Heil J., Zajic S., Brangan A., Dairo O., Smith G., Morales-Scheihing D., Buono R.J., Ferraro T.N., Haroz R. (2022). Patient Perceptions and Potential Utility of Pharmacogenetic Testing in Chronic Pain Management and Opioid Use Disorder in the Camden Opioid Research Initiative. Pharmaceutics.

[9] Lanting P., Drenth E., Boven L., van Hoek A., Hijlkema A., Poot E., van der Vries G., Schoevers R., Horwitz E., Gans R. (2020). Practical Barriers and Facilitators Experienced by Patients, Pharmacists and Physicians to the Implementation of Pharmacogenomic Screening in Dutch Outpatient Hospital Care—An Explorative Pilot Study. J. Pers. Med..

[10] Lee G., Varughese L.A., Conway L., Stojinski C., Ashokkumar S., Monono K., Matthai W., Kolansky D.M., Giri J., Tuteja S. (2022). Attitudes toward pharmacogenetics in patients undergoing CYP2C19 testing following percutaneous coronary intervention. Pers. Med..

[11] Allen J.D., Pittenger A.L., Bishop J.R. (2022). A Scoping Review of Attitudes and Experiences with Pharmacogenomic Testing among Patients and the General Public: Implications for Patient Counseling. J. Pers. Med..

[12] Schmidlen T., Sturm A.C., Scheinfeldt L.B. (2020). Pharmacogenomic (PGx) counseling: Exploring participant questions about PGx test results. J. Pers. Med..

[13] Pacyna J.E., Radecki Breitkopf C., Jenkins S.M., Sutton E.J., Horrow C., Kullo I.J., Sharp R.R. (2019). Should pretest genetic counselling be required for patients pursuing genomic sequencing? Results from a survey of participants in a large genomic implementation study. J. Med. Genet..

[14] Sutton E.J., Kullo I.J., Sharp R.R. (2018). Making pretest genomic counseling optional: Lessons from the RAVE study. Genet. Med. Off. J. Am. Coll. Med. Genet..

[15] Bielinski S.J., St Sauver J.L., Olson J.E., Larson N.B., Black J.L., Scherer S.E., Bernard M.E., Boerwinkle E., Borah B.J., Caraballo P.J. (2020). Cohort Profile: The Right Drug, Right Dose, Right Time: Using Genomic Data to Individualize Treatment Protocol (RIGHT Protocol). Int. J. Epidemiol..

[16] Wang L., Scherer S.E., Bielinski S.J., Muzny D.M., Jones L.A., Black J.L., Moyer A.M., Giri J., Sharp R.R., Matey E.T. (2022). Implementation of preemptive DNA sequence-based pharmacogenomics testing across a large academic medical center: The Mayo-Baylor RIGHT 10K Study. Genet. Med. Off. J. Am. Coll. Med. Genet..

[17] OneOme. https://oneome.com/.

[18] DeSalvo K.B., Fan V.S., McDonell M.B., Fihn S.D. (2005). Predicting mortality and healthcare utilization with a single question. Health Serv. Res..

[19] Chew L.D., Griffin J.M., Partin M.R., Noorbaloochi S., Grill J.P., Snyder A., Bradley K.A., Nugent S.M., Baines A.D., Vanryn M. (2008). Validation of screening questions for limited health literacy in a large VA outpatient population. J. Gen. Intern. Med..

[20] Arwood M.J., Chumnumwat S., Cavallari L.H., Nutescu E.A., Duarte J.D. (2016). Implementing Pharmacogenomics at Your Institution: Establishment and Overcoming Implementation Challenges. Clin. Transl. Sci..

[21] Rosenman M.B., Decker B., Levy K.D., Holmes A.M., Pratt V.M., Eadon M.T. (2017). Lessons Learned When Introducing Pharmacogenomic Panel Testing into Clinical Practice. Value Health.

[22] Lee Y.M., Manzoor B.S., Cavallari L.H., Nutescu E.A. (2018). Facilitators and Barriers to the Adoption of Pharmacogenetic Testing in an Inner-City Population. Pharmacotherapy.

[23] Rafi I., Crinson I., Dawes M., Rafi D., Pirmohamed M., Walter F.M. (2020). The implementation of pharmacogenomics into UK general practice: A qualitative study exploring barriers, challenges and opportunities. J Community Genet.

[24] Lachance K., Korol S., O’Meara E., Ducharme A., Racine N., Liszkowski M., Rouleau J.L., Pelletier G.B., Carrier M., White M. (2015). Opinions, hopes and concerns regarding pharmacogenomics: A comparison of healthy individuals, heart failure patients and heart transplant recipients. Pharmacogenomics J..

[25] Gawronski B.E., Cicali E.J., McDonough C.W., Cottler L.B., Duarte J.D. (2023). Exploring perceptions, knowledge, and attitudes regarding pharmacogenetic testing in the medically underserved. Front. Genet..

[26] Lemke A.A., Amendola L.M., Thompson J., Dunnenberger H.M., Kuchta K., Wang C., Dilzell-Yu K., Hulick P.J. (2021). Patient-Reported Outcomes and Experiences with Population Genetic Testing Offered Through a Primary Care Network. Genet. Test. Mol. Biomark..

[27] Olson J.E., Rohrer Vitek C.R., Bell E.J., McGree M.E., Jacobson D.J., St Sauver J.L., Caraballo P.J., Griffin J.M., Roger V.L., Bielinski S.J. (2017). Participant-perceived understanding and perspectives on pharmacogenomics: The Mayo Clinic RIGHT protocol (Right Drug, Right Dose, Right Time). Genet. Med. Off. J. Am. Coll. Med. Genet..

[28] Mills R., Ensinger M., Callanan N., Haga S.B. (2017). Development and Initial Assessment of a Patient Education Video about Pharmacogenetics. J. Pers. Med..

[29] Truong T.M., Lipschultz E., Danahey K., Schierer E., Ratain M.J., O’Donnell P.H. (2020). Assessment of Patient Knowledge and Perceptions of Pharmacogenomics Before and After Using a Mock Results Patient Web Portal. Clin. Transl. Sci..

[30] Asiedu G.B., Finney Rutten L.J., Agunwamba A., Bielinski S.J., St Sauver J.L., Olson J.E., Rohrer Vitek C.R. (2020). An assessment of patient perspectives on pharmacogenomics educational materials. Pharmacogenomics.

[31] Pacyna J.E., Shaibi G.Q., Lee A., Byrne J.O., Cuellar I., Sutton E.J., Hernandez V., Lindor N.M., Singh D., Kullo I.J. (2021). Increasing access to individualized medicine: A matched-cohort study examining Latino participant experiences of genomic screening. Genet. Med. Off. J. Am. Coll. Med. Genet..

